# Supplementary material for: Patterns and Predictors of Low-Calorie Sweetener Consumption during Pregnancy: Findings from a National Survey
Source: Nutrients. 2023 Sep 28;15(19):4196. doi: 10.3390/nu15194196 (PMC10574556; doi:10.3390/nu15194196)
Supplement: Supplementary file 1 [file nutrients-15-04196-s001.zip › nutrients-2621457-supplementary.pdf]

Patterns and predictors of low-calorie sweetener consumption during pregnancy: findings from a national survey

Bereket Gebremichael[1,2,3] Zohra S Lassi[2,4] Mumtaz Begum[2,5] Murthy Mittinty[6]
Shao Jia Zhou[1,2*]

Affiliations

1. Department of Food and Nutrition, School of Agriculture, Food and Wine, The University of Adelaide, Adelaide, Australia
2. Robinson Research Institute, The University of Adelaide, Adelaide, Australia
3. College of Health Science, Addis Ababa University, Addis Ababa, Ethiopia
4. School of Public Health, Faculty of Health and Medical Sciences, The University of Adelaide
5. Adelaide Medical School, The University of Adelaide, Adelaide, Australia
6. College of Medicine and Public Health, Flinders University, Adelaide, Australia

Table S1. Checklist for Reporting Results of Internet E-Surveys (CHERRIES)

Item category	*Checklist Item*	*Description*	*Page Number*
**Design**	Describe survey design	Pregnant women aged 18-50 in Australia.	4
**Ethics**	IRB approval	The study was reviewed by the University of Adelaide Human Research Ethics Committee, and the necessary ethical clearance was obtained (Ethics approval number H-2022-040).	20
	Informed consent	Participant information sheet and consent form was embedded in the survey preamble. The survey preamble contained an introduction to the survey, its objective, the range of question asked, how to respond to the questions, the volunteer nature of the survey, their right to withdraw from the survey without any consequence, anonymity, and contact details of the investigator(s). At the end of the survey preamble, eligible participants were given two choices, 'Yes, I agree to participate' and 'No'. Participants were advised that by clicking the 'Yes, I agree to participate' choice, they indicated that they read and understood the information provided in the preamble and consent to participate in this survey.	20
	Data protection	Qualtrics holds some personal information of their panel members (consumers), which could be used by Qualtrics to make the initial contact to gauge their interest to participate in a study. The researchers do not have access to the personal information of respondents of the survey. The data collected in the survey are anonymous.	4
**Development and pretesting**	Development and testing	The survey was developed by the research team though literature review and consultation of experts in the field. Usability and technical functionality of the electronic questionnaire was pre-tested by researchers and other invited child-bearing aged women who had no or limited knowledge of the subject matter and were not part of the study.	5
**Recruitment process**	Open survey versus closed survey	Open survey	4
	Contact mode	The consumer panel provider Qualtrics contacted potential participants. The researcher had no role in recruiting participants.	4

	Advertising the survey	Qualtrics used various methods to initially contact participants, this included; 1) potential participants in the target population were sent an email invitation informing them about a survey which is for research purposes only, how long the survey would take, and what incentives were available, 2) through posting the survey on their website to potential participants who were likely to qualify for upon signing into a panel portal, 3) other invitation methods including in-app notifications and SMS notifications.	4
**Survey administration**	Web/E-mail	Posted on a Web site of the panel provider and invitation email were also sent to potential participants	4
	Context	Qualtrics online survey platform. Qualtrics is a reputable panel provider that is commonly used by researchers to conduct online surveys.	4
	Mandatory/voluntary	Voluntary survey	4
	Incentives	Qualtrics provides an incentive to their survey respondents based on the length of the survey and the difficulty of recruiting the target population amongst other factors. For surveys like this, the incentive is approximately $5 in the monetary term. The specific rewards vary and may include cash, airline miles, gift cards, redeemable points, charitable donations, sweepstakes entrance, and vouchers.	NA
	Time/Date	September to October, 2022	2
	Randomization of items or questionnaires	No	NA
	Adaptive questioning	Yes,<br>Some questions were only conditionally displayed based on responses to prior response.	NA
	Number of Items	24 questions	NA
	Number of screens (pages)	6 pages if completed on a computer and 8 pages in using mobile phone.	NA
	Completeness check	Approximately 80% of the questions were mandatory items. Respondent wouldn’t be able to move to the next page without answering those questions.	NA
	Review step	Respondents were able to review and change their answers before clicking the submit button. Once it has been submitted, they were not able to make any further changes.	NA

**Response rates**	Unique site visitor	Not reported	NA
	View rate (Ratio of unique survey visitors/unique site visitors)	Not reported	NA
	Participation rate (Ratio of unique visitors who agreed to participate/unique first survey page visitors)	1273/1284	Supplementary Figure 1
	Completion rate (Ratio of users who finished the survey/users who agreed to participate)	560/1273	Supplementary Figure 1
**Preventing multiple entries from same individual**	Cookies used	No cookies were used to assign a unique user identifier to each client computer/phone.	NA
	IP check	IP address of the client computer/phone was used to identify potential duplicate entries from the same user. Duplicate database entries having the same IP address were eliminated before analysis. The first entry was kept for analysis.	NA
	Log file analysis	Not performed	NA
	Registration	Prior registration to this survey was not necessary participate.	NA
**Analysis**	Handling of incomplete questionnaires	We only analyzed completed questionnaires.	NA
	Questionnaires submitted with an atypical timestamp	Responses that were completed in four minutes or less are excluded from the analysis. Four minutes was the mean time to complete the survey during the pretest.	NA
	Statistical correction	We didn’t employ any statistical correction as it was not necessary.	NA

Table S2. Consumption of food group containing low calorie sweetener

Food Group	Consumption Frequency

	Never n (%)	<Once per Week n (%)	Once per Week n (%)	2-3 Times per Week n (%)	4-6 times per week n (%)	Daily n (%)
Soft drink	118 (27.96)	87(20.62)	60(14.22)	84(19.91)	33(7.82)	40(9.48)
Cordial drink	236 (55.92)	67(15.88)	38(9.00)	59(13.98)	8(1.90)	14(3.32)
Yoghurt or mousse	262(62.09)	58(13.74)	42(9.95)	46(10.90)	8(1.90)	6(1.42)
Chewing gum	171(40.52)	94(22.27)	61(14.45)	60(14.22)	22(5.21)	14(3.32)
Jelly/pudding/jam	320(75.83)	49(11.61)	24(5.69)	25(5.92)	2 (0.47)	2 (0.47)
Flavoured milk/chocolate drink	240(56.87)	89(21.09)	48(11.37)	37(8.77)	6(1.42)	2 (0.47)
Ice-cream	276(65.40)	73(17.30)	35(8.29)	33(7.82)	4(0.95)	1(0.24)
Energy drink	353(83.65)	34(8.06)	20(4.74)	10(2.37)	4(0.95)	1(0.24)
Protein drink	352(83.41)	27(6.40)	21(4.98)	15(3.55)	4(0.95)	3(0.71)
Iced tea	261(61.85)	96(22.75)	33(7.82)	24(5.69)	4(0.95)	4(0.95)
Biscuits	201(47.63)	89(21.09)	66(15.64)	53(12.56)	5(1.18)	8(1.90)
Cooking/baking	276(65.40)	57(13.51)	48(11.37)	27(6.40)	6(1.42)	8(1.90)

Diet soft drink include diet flavoured mineral water, diet cordial drinks include fruit flavoured or powdered drink mixes, chewing gum or lollies include chocolate or confectionary, Ice-tea include artificially sweetened tea like Fuze iced-tea or Lipton Ice-tea, Cooking & baking include Artificial sweeteners in cooking/baking, tea or coffee.

Table S3. Participant overall characteristics based on any low-calorie sweetener consumption [a].

Characteristics	Category	LCS Consumption		P-Value
		No (n=21)	Yes (n=401)	
Age		30.5±3.5	30±4.7	0.61
BMI		26±5.0	27.2±6.6	0.43
Educational level	Secondary	23.8	19.2	
	Post-secondary but no tertiary	14.3	36.2	
	Tertiary	61.9	44.6	0.12
Employment status	Not working	28.6	17.7	
	Working	71.4	82.3	0.21
General medical condition	No	71.4	80.5	
	Yes, DM	-	4.2	
	Yes, other	28.6	15.2	0.19
Parity	Primi/Zero	33.3	40.4	
	One	52.4	41.6	
	Two or more	14.3	17.9	0.62
Gestation age	First trimester	9.5	19.7	
	Second trimester	38.1	30.9	
	Third trimester	52.4	49.4	0.49
Pregnancy complication	None	57.1	67.3	
	Hypertension, Anaemia & other	28.6	17.4	
	GDM	14.3	10.2	0.62
Pre-pregnancy supplement use	Did not use	57.1	44.9	
	Used but do not contain I and F	14.3	9.5	

	Use I & F containing supplement	28.6	45.6	0.30
Pregnancy supplement use	Do not use	14.3	8.2	
	Use but do not contain I and F	28.6	8.7	
	Use I & F containing supp	57.1	83.0	0.005
Pre-pregnancy Alcohol use	No	38.1	32.4	
	Yes	61.9	67.6	0.59
Pregnancy alcohol use	No	95.2	94.3	
	Yes	4.8	5.7	0.72
Smoking habit	Never smoker	90.4	91.8	
	Past smoker	4.8	7.0	
	Current smoker	4.8	1.2	0.39
Moderate physical activity during current pregnancy	<= 2 times per week	95.2	75.8	
	3-4 times per week	-	19.2	
	>=5 times per week	4.8	5.0	0.08
IRSAD	First (Disadvantaged)	20.0	20.2	
	Second	40.0	19.4	
	Third	25.0	20.2	
	Fourth	15.0	19.1	
	Fifth (Advantaged)	-	21.0	0.07

[a] Data are Mean±SD for continuous variables and frequency (%) for categorical variable. We used T-test to compare mean between groups for numeric variables and chi2 test to compare proportion difference for categorical variable. DM: diabetes mellitus; GDM: gestational diabetes mellitus; I: iodine; F: folate; LCS: low-calorie sweetener: IRSAD: Index of Relative Socio-economic Advantage and Disadvantage.

Table S4. Adherence to the recommended daily serve size based on the Australian dietary guideline for the five food groups.

**Food Group**	**Mean (SD) Intake**	**Recommended Number of Daily serves**	**Meeting the Requirement n (%)**
Vegetable and legumes/beans (n=418)	2.1(1.3)	5	18(4.27)
Fruit (n=421)	1.6(1.0)	2	212(50.24)
Lean meats and poultry, fish, egg (n=420)	1.7(3.9)	3.5	12(2.84)
Milk and milk product	1.5(1.0)	2.5	62(14.69)
Grain (cereal) (n=421)	1.8(1.2)	8.5	-

Vegetable: recommended serves per day for pregnant women is 5 serves. A standard serve of vegetables is about 75g (100-350kJ). Example, ½ cup cooked green or orange vegetables (for example, broccoli, spinach, carrots, or pumpkin); ½ cup cooked, dried or canned beans, peas or lentils; 1 cup green leafy or raw salad vegetables; ½ cup sweet corn; ½ medium potato or other starchy vegetables (sweet potato, taro or cassava); 1 medium tomato.

Fruit: recommended serves per day for pregnant women is 2 serves A standard serve of fruit is about 150g (350kJ). Example, 1 medium apple, banana, orange, or pear; 2 small apricots, kiwi fruits or plums; 1 cup diced or canned fruit (with no added sugar)

Meat and poultry: recommended serves per day for pregnant women is 3.5 serves a standard serve is 500-600kJ. Example: 65g cooked lean meats such as beef, lamb, veal, pork, goat or kangaroo (about 90–100g raw)* 80g cooked lean poultry such as chicken or turkey (100g raw) 100g cooked fish fillet (about 115g raw weight) or one small can of fish 2 large (120g) eggs 1 cup (150g) cooked or canned legumes/beans such as lentils, chick peas or split peas (preferably with no added salt) 170g tofu 30g nuts, seeds, peanut or almond butter or tahini or other nut or seed paste (no added salt)

Milk, milk product and alternatives: recommended serves per day for pregnant women is 2.5 serves A standard serve is 500-600kJ. Example of serve size, 1 cup (250ml) fresh, UHT long life, reconstituted powdered milk or buttermilk; ½ cup (120ml) evaporated milk; 2 slices (40g) or 4 x 3 x 2cm cube (40g) of hard cheese, such as cheddar; ¾ cup (200g) yoghurt; 1 cup (250ml) soy, rice or other cereal drink with at least 100mg of added calcium per 100ml.

Grain (cereal): recommended serves per day for pregnant women is 8.5 serves A standard serve is 500kJ. Example, 1 slice (40g) bread; ½ medium (40g) roll or flat bread; ½ cup (75–120g) cooked rice, pasta, noodles, barley, buckwheat, semolina, polenta, bulgur or quinoa; ½ cup (120g) cooked porridge; ²/³ cup (30g) wheat cereal flakes; ¼ cup (30g) muesli; 3 (35g) crispbreads; 1 (60g) crumpet; 1 small (35g) English muffin or scone [1].

Table S5. Multinomial regression of factors predicting latent class membership of class two and three in relative to class one (*n=416*)

		Model One				Model Two				Model Three			
**Variables**	**Category**	**Moderate Consumption**		**Habitual Consumption**		**Moderate Consumption**		**Habitual Consumption**		**Moderate Consumption**		**Habitual Consumption**	
		**aRR R**	**95% CI**	**aRR R**	**95% CI**	**aRR R**	**95% CI**	**aRR R**	**95% CI**	**aRR R**	**95% CI**	**aRR R**	**95% CI**
Age [a]		0.94	0.90-0.99	0.90	0.84-0.98	0.94	0.90-0.99	0.91	0.84-0.98	0.93	0.88-0.98	0.92	0.84- 1.01
Employment condition	Not working	Ref				Ref				Ref			
	Working	2.63	1.48-4.69	2.38	0.85-6.63	2.69	1.48-4.88	2.66	0.92-7.70	3.25	1.67-6.33	2.71	0.85-8.60
Educational level	Secondary	Ref				Ref				Ref			
	Above secondary but not tertiary	1.27	0.71-2.28	0.95	0.33-2.68	1.33	0.74-2.41	0.92	0.32-2.66	1.29	0.69-2.41	0.79	0.25-2.50
	Degree and above	0.65	0.36-1.16	1.42	0.55-3.69	0.68	0.38-1.23	1.23	0.46-3.32	0.77	0.41-1.45	1.37	0.46-4.07
General medical condition	No					Ref				Ref			
	Yes-DM					2.54	0.76-8.43	5.09	0.97-26.79	2.14	0.56-8.16	3.06	0.45-20.54
	Yes, other					0.57	0.31-1.03	0.32	0.09-1.12	0.45	0.23-0.88	0.20	0.05-0.80
Pre pregnancy alcohol use	No					Ref				Ref			
	Yes					0.83	0.52-1.32	0.48	0.23-0.96	0.81	0.49-1.32	0.65	0.29-1.45
BMI [a]						0.99	0.96-1.02	0.92	0.86-0.98	0.99	0.95-1.02	0.89	0.83-0.97
Percentage of adherence to ADG [a]										0.98	0.97-0.99	0.98	0.95-1.00
SSB consumption	No or less frequently									Ref			
	Frequently									1.34	0.82-2.19	3.17	1.39-7.21
Moderate physical activity during pregnancy	No or < 2 times per week									Ref			
	3-4 times per week									1.04	0.57-1.91	1.98	0.79-4.92
	>= 5 times per week									1.74	0.58-5.18	3.92	0.93-16.51
Pregnancy supplement use	Not at all									Ref			
	Use, don't contain I&F									2.29	0.76-6.85	4.07	0.54-30.29
	Use, contain I & F									2.71	1.13-6.47	3.77	0.80-17.74
Parity	Primi/ Zero									Ref			

	One									0.90	0.54-1.49	0.89	0.37-2.13
	Two or more									1.93	0.96-3.89	3.06	0.94-9.93
Pregnancy duration	First trimester									Ref			
	Second trimester									0.96	0.49-1.87	0.85	0.30-2.38
	Third trimester									0.80	0.43-1.49	0.38	0.14-1.04
Pregnancy complication	None									Ref			
	Hypertension, anaemia & other									1.73	0.98-3.06	3.11	1.26-7.67
	GDM									1.27	0.55-2.95	3.53	1.03-12.10
LCS safety concern on the baby	No									Ref			
	Not sure									0.85	0.50-1.44	0.56	0.22-1.43
	Yes									0.61	0.34-1.10	0.57	0.22-1.51

Model one; adjusted for sociodemographic factors, Model two; Model one and additionally adjusted for pre-pregnancy health and lifestyle factors, Model three; Model two and additionally adjusted for pregnancy condition and LCS safety concern.

[a] continuous variable BMI Body Mass Index, F Folate, GDM Gestational Diabetes Mellites, I Iodine, LCS Low Calorie Sweetener, SSB Sugar Sweetened Beverage

Ordinal logistic regression

Respondents were asked to report the frequency of consumption for each of the food twelve-food group on a six-point scale (Never, <1 per week, once per week, 2-4 times per week, 4-6 times per week, and daily). To determine LCS consumption frequency per week, first we re-coded the frequency interval into number of consumptions as follows Never=0, <1 per week=0.5/week, 1 per week= 1, 2-4 per week=3, 4-6 per week=5, and daily=7. Then we computed the total frequency of LCS consumption per week by adding scores from each of the twelve-food group. To fit ordinal logistic regression model, the total LCS consumption frequency was divided into tercile.

Table S6. Predictors of frequent consumption of LCS among pregnant women in Australia (*n=416*)

Variables	Category	Model 1		Model 2		Model 3	
		**AOR**	**95% CI**	**AOR**	**95% CI**	**AOR**	**95% CI**
Age [a]		**0.97**	**0.93-1.00**	0.96	0.92-0.99	0.98	0.94-1.03
Employment condition	Not working	Ref					
	Working	**1.92**	**1.19-3.10**	**1.85**	**1.11-3.06**	**1.85**	**1.07-3.20**
Educational level	Secondary	Ref					
	Above secondary but not tertiary	1.32	0.79-2.18	1.36	0.81-2.28	1.28	0.74-2.22
	Degree and above	0.79	0.49-1.31	0.82	0.49-1.38	0.88	0.51-1.54
Pre-pregnancy alcohol use	No			Ref			
	Yes			0.57	0.38-0.85	**0.56**	**0.37-0.86**
General medical condition	No			Ref			
	Yes-DM			**6.16**	**1.89-20.03**	**5.90**	**1.55-22.46**
	Yes, other			**0.54**	**0.33-0.95**	**0.42**	**0.24-0.74**
BMI [a]						0.99	0.97-1.03
Percentage of adherence to ADG [a]						0.99	0.98-1.01
SSB consumption	No or less frequently					Ref	
	Frequently					**2.40**	**1.57-3.65**
Moderate physical activity during pregnancy	<=2 times per week					Ref	
	3-4 times per week					1.20	0.72-2.00
	>= 5 times per week					1.58	0.65-3.81
Pregnancy supplement use	Not at all					Ref	
	Use, don't contain I&F					1.41	0.52-3.87
	Use, contain I & F					**2.50**	**1.16-5.38**

Parity	Primi/ Zero					Ref	
	One					0.85	0.55-1.31
	Two or more					1.14	0.62-2.12
Pregnancy duration	First trimester					Ref	
	Second trimester					**0.53**	**0.30-0.94**
	Third trimester					**0.47**	**0.28-0.80**
Pregnancy complication	None					Ref	
	Hypertension, anaemia & other					**1.73**	**1.06-2.80**
	GDM					1.45	0.69-2.99
Safety concern towards the baby	No					Ref	
	Not sure					0.64	0.41-0.1.01
	Yes					**0.45**	**0.28-0.74**

Model one; adjusted for sociodemographic factors, Model two; Model one and additionally adjusted for lifestyle and dietary factor, Model three; Model two and additionally adjusted for pregnancy condition and safety related factors.

[a] *continuous variable, BMI Body Mass Index, F Folate, GDM Gestational Diabetes Mellites, I Iodine, LCS Low Calorie Sweetener, SSB Sugar Sweetened Beverage*

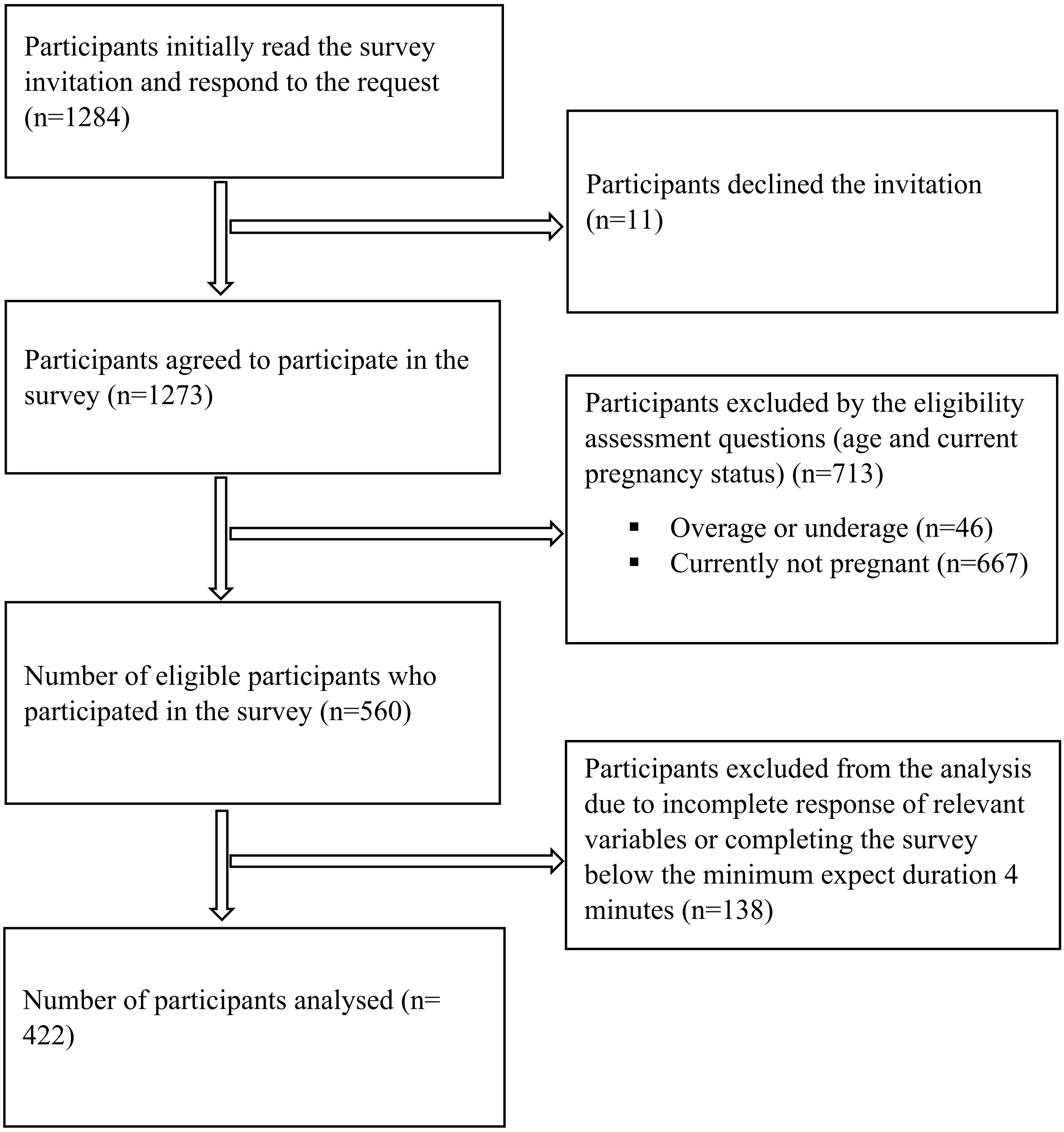

Figure S1. Flow diagram of the selection of survey participants

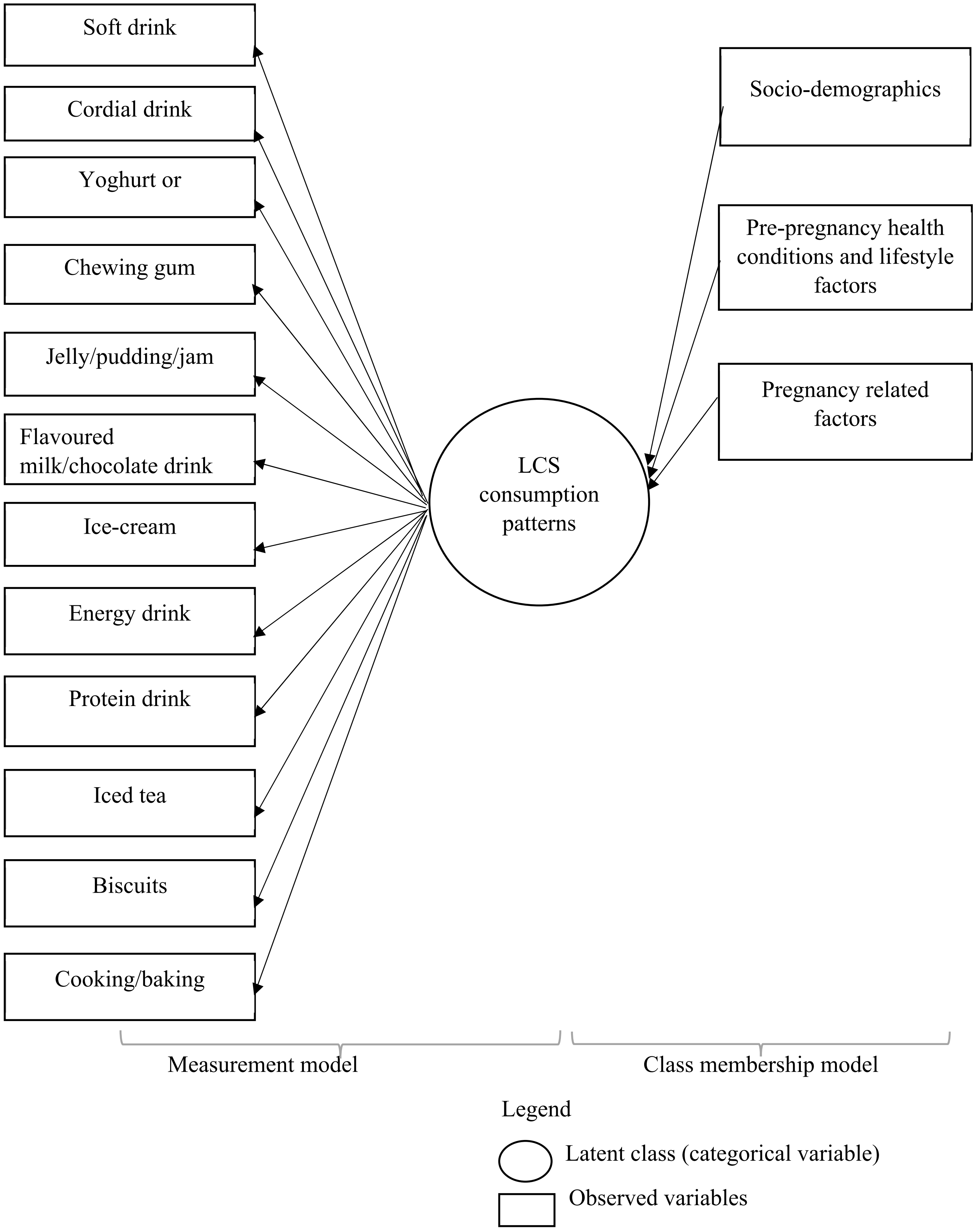

Figure S2. Graphical presentation of latent class analysis with covariates: modified from previous study [2]

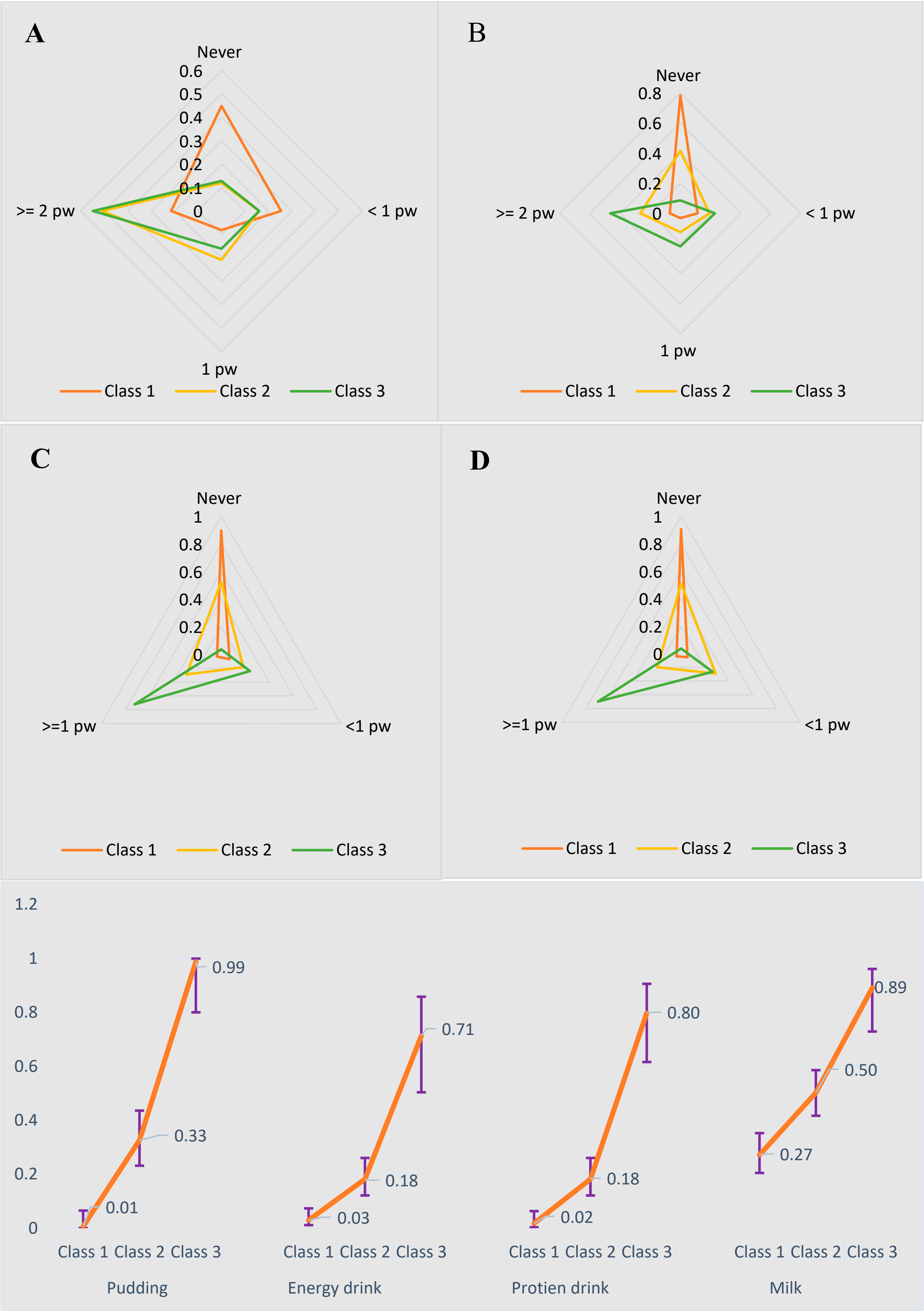

Figure S3. Estimated response (conditional) probabilities of selected low-calorie sweetened food and drink consumption in each latent class using a radar plot and line graph.

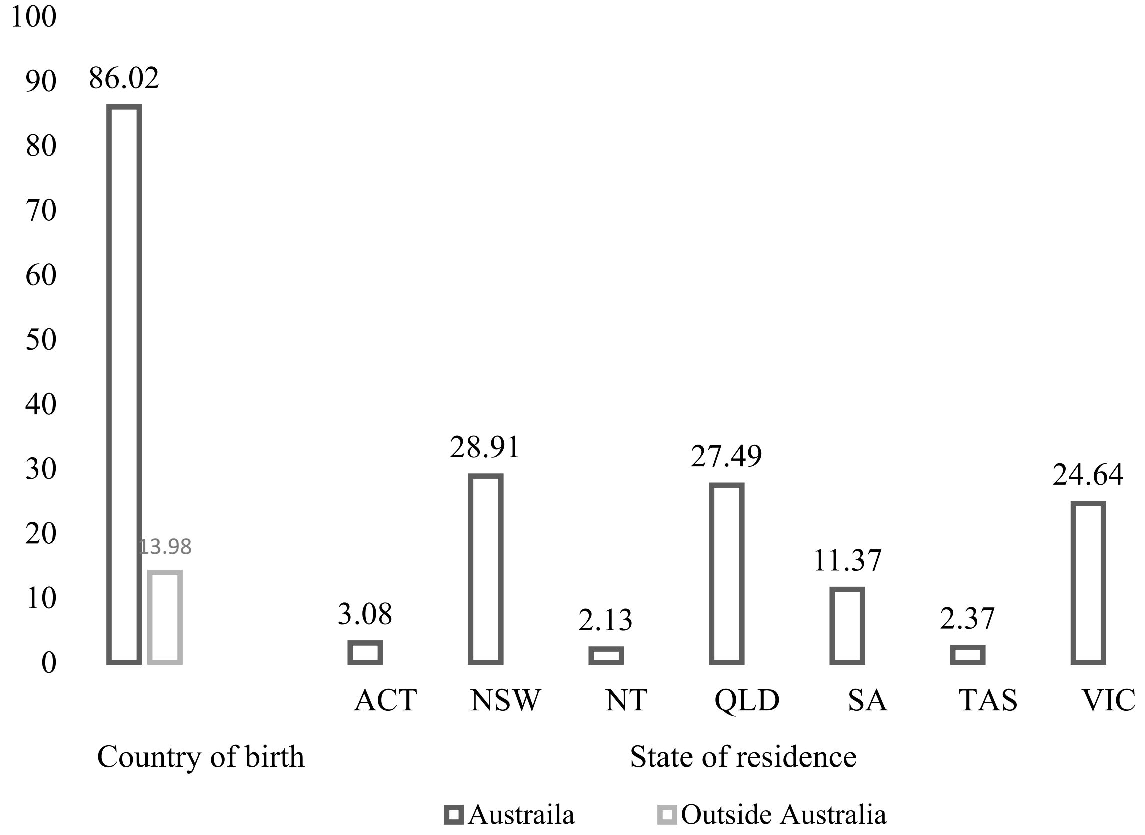

Figure S4. Participants distribution by country of birth and state of residence

Country of birth outside Australia include New Zealand, England, Sri Lanka, USA, Malaysia, India, Philippines, and South Africa

Reference

1. National Health and Medical Research Council. *Australian Dietary Guidelines*; National Health and Medical Research Council: 2013.
2. Molin, E.; Mokhtarian, P.; Kroesen, M. Multimodal travel groups and attitudes: A latent class cluster analysis of Dutch travelers. *Transportation Research Part A: Policy and Practice* **2016**, *83*, 14-29, doi:https://doi.org/10.1016/j.tra.2015.11.001.
